# Theranostic digital twins: Concept, framework and roadmap towards personalized radiopharmaceutical therapies

**DOI:** 10.7150/thno.93973

**Published:** 2024-05-27

**Authors:** Hamid Abdollahi, Fereshteh Yousefirizi, Isaac Shiri, Julia Brosch-Lenz, Elahe Mollaheydar, Ali Fele-Paranj, Kuangyu Shi, Habib Zaidi, Ian Alberts, Madjid Soltani, Carlos Uribe, Babak Saboury, Arman Rahmim

**Affiliations:** 1 Department of Radiology, University of British Columbia, Vancouver, Canada.; 2 Department of Integrative Oncology, BC Cancer Research Institute, Vancouver, Canada.; 3 Division of Nuclear Medicine and Molecular Imaging, Geneva University Hospital, Geneva, Switzerland.; 4 Department of Cardiology, University Hospital Bern, Switzerland.; 5 Department of Nuclear Medicine, University Hospital, LMU Munich, Munich, Germany.; 6 Department of Biomedical Engineering, University of British Columbia, Vancouver, Canada.; 7 Department of Mathematics, University of British Columbia, Vancouver, Canada.; 8 Department of Nuclear Medicine, Inselspital, Bern University Hospital, University of Bern, Bern, Switzerland.; 9 Department of Informatics, Technical University of Munich, Munich, Germany.; 10 Department of Nuclear Medicine and Molecular Imaging, University of Groningen, University Medical Center Groningen, Groningen, Netherlands.; 11 Department of Nuclear Medicine, University of Southern Denmark, Odense, Denmark.; 12 University Research and Innovation Center, Óbuda University, Budapest, Hungary.; 13 Department of Molecular Imaging and Therapy, BC Cancer, Vancouver, Canada.; 14 Department of Mechanical Engineering, K. N. Toosi University of Technology, Tehran, Iran.; 15 Department of Electrical and Computer Engineering, University of Waterloo, ON, Canada.; 16 Department of Radiology and Imaging Sciences, Clinical Center, National Institutes of Health, Bethesda, USA.; 17 Department of Physics and Astronomy, University of British Columbia, Vancouver, Canada.

**Keywords:** Digital twins, Radiopharmaceutical therapies, Theranostics, Roadmap, Personalized therapy, Precision Medicine

## Abstract

Radiopharmaceutical therapy (RPT) is a rapidly developing field of nuclear medicine, with several RPTs already well established in the treatment of several different types of cancers. However, the current approaches to RPTs often follow a somewhat inflexible “one size fits all” paradigm, where patients are administered the same amount of radioactivity per cycle regardless of their individual characteristics and features. This approach fails to consider inter-patient variations in radiopharmacokinetics, radiation biology, and immunological factors, which can significantly impact treatment outcomes. To address this limitation, we propose the development of theranostic digital twins (TDTs) to personalize RPTs based on actual patient data. Our proposed roadmap outlines the steps needed to create and refine TDTs that can optimize radiation dose to tumors while minimizing toxicity to organs at risk. The TDT models incorporate physiologically-based radiopharmacokinetic (PBRPK) models, which are additionally linked to a radiobiological optimizer and an immunological modulator, taking into account factors that influence RPT response. By using TDT models, we envisage the ability to perform virtual clinical trials, selecting therapies towards improved treatment outcomes while minimizing risks associated with secondary effects. This framework could empower practitioners to ultimately develop tailored RPT solutions for subgroups and individual patients, thus improving the precision, accuracy, and efficacy of treatments while minimizing risks to patients. By incorporating TDT models into RPTs, we can pave the way for a new era of precision medicine in cancer treatment**.**

## 1. Introduction

Cancer is a complex disease and a leading cause of death worldwide 1. Despite recent advancements in cancer therapy, many treatments are often not fully effective for different types of cancers, underscoring the need for further research and development efforts 2. In addition, current treatments often lack true personalization and can follow an empirical “one size fits all” approach 3. Meanwhile, cancer complexities, such as heterogeneities and microenvironment-related characteristics are critical factors that are not fully considered in current cancer treatment strategies (4,5). Furthermore, the potential for detriment to healthy tissues remains a major concern in most of these therapies, which limits treatment dose escalation.

Radiopharmaceutical therapy (RPT) is a long-established tool in the armamentarium for the treatment of numerous cancers and most recently has gained a pivotal role in the management of advanced prostate cancer (PCa) and neuroendocrine tumors (NETs) 4-6. RPT, although not a recent innovation, stands as a firmly established and evidence-based approach in the treatment of various diseases. Its applications span the management of differentiated thyroid cancer, benign thyroid diseases, synovial ablation in conditions like pigmented villonodular synovitis (PVNS), and addressing refractory bone pain in breast and prostate cancer 7. Additionally, RPT is recognized for its efficacy in the ablation of hepatocellular carcinoma (HCC) and liver metastases through Y-90-SIRT, with endorsement from guidelines in many instances 8. Beyond these well-established uses, emerging therapies have gained marketing approval, notably for squamous cell cancers and a diverse array of investigational theragnostic compounds.

It is crucial to recognize the extensive history of RPT, encompassing both enduring practices and evolving therapeutic landscapes. While some older treatments like P-32 for Polycythemia Vera, MIBG therapy, and CD-20 mABs for Lymphoma may have lost favor, they demonstrate the rich history of theranostic approaches in nuclear medicine 9. Buoyed by this success, there are ongoing research efforts to develop RPTs in other entities, for example in the management of myeloma and plasma-dyscrasia 10, with an even greater role for RPT expected in the coming years. However, despite legal mandates calling for personalized dosimetry, such as the European Council directives enshrined in the Euratom treaty 11, personalized dosimetry is seldom performed. It is therefore a major shortcoming of RPT that patients most commonly receive a standard and empirical activity (e.g., 7.4 GBq) 12. For example, Lu-177-Lutathera was approved with a standard administration dosage (7.4 GBq every 8 weeks for a total of 4 administrations) without any requirements for personalized dosimetry 13. Furthermore, in the peptide receptor radionuclide therapy (PRRT) of NETs, the maximum tolerable dose to limiting organs often is not reached. With the majority of patients eventually progressing, there is substantial risk that the use of empirical “one size fits all” approaches leads to undertreatment in many patients 14.

As a prime example of cutting-edge RPTs, consider their remarkable efficacy in treating advanced prostate cancer (PCa), notably metastatic castration-resistant prostate cancer (mCRPCa) using therapies such as Lu-177-PSMA 15. This targeted approach, utilizing Lu-177-PSMA, capitalizes on the overexpression of prostate-specific membrane antigen (PSMA) receptors on prostate cancer cells 16. PSMA serves as a critical biomarker for disease progression, playing a pivotal role in guiding treatment decisions and monitoring response to therapy. In addition to its role as a biomarker, PSMA is implicated in various aspects of prostate cancer biology, including tumor growth, metastasis, and disease aggressiveness 17. Its overexpression on cancer cells makes it an attractive therapeutic target, enabling selective targeting while minimizing off-target effects on healthy tissues. Moreover, PSMA's utility extends beyond treatment; it serves as a valuable diagnostic tool for identifying and monitoring prostate cancer progression, aiding in disease staging and prognosis assessment.

Despite their remarkable success, current RPT approaches face a critical challenge: the lack of specific and optimized protocols for individualized therapy. Remarkably, all mCRPCa patients are subjected to the same standardized treatment regimen, underscoring a glaring gap in personalized medicine within this field 18. This “one size fits all” paradigm increases the risk of both overdosing and underdosing, and reduces the likelihood of achieving optimal tumor control while mitigating normal tissue complications 19. In fact, in current non-personalized paradigms, the range of absorbed doses to organs-at-risk (e.g. salivary glands) can span an order of magnitude 20 resulting in conservative schemes in practice where many of patients are undertreated. There is thus a need for innovative approaches to optimize therapies while mitigating the side effects of treatment and enhancing the well-being of patients undergoing RPTs. Finding effective solutions including to manage toxicity levels is essential for maintaining a high quality of life throughout the treatment process 15. In other areas of oncology, substantial progress toward the realization of personalized treatments has been made 21. By utilizing advanced technologies such as omics-based approaches (e.g., genomics, radiomics) and artificial intelligence (AI), it is now possible to identify unique biomarkers that can help predict treatment response and guide personalized therapies 22,23. As a result, promising outcomes have been observed in clinical trials and real-world settings, suggesting that personalized treatments have the potential to greatly improve patient outcomes and quality of life 24 and which we are confident will also make a positive impact if integrated into RPT.

**Figure 1:**
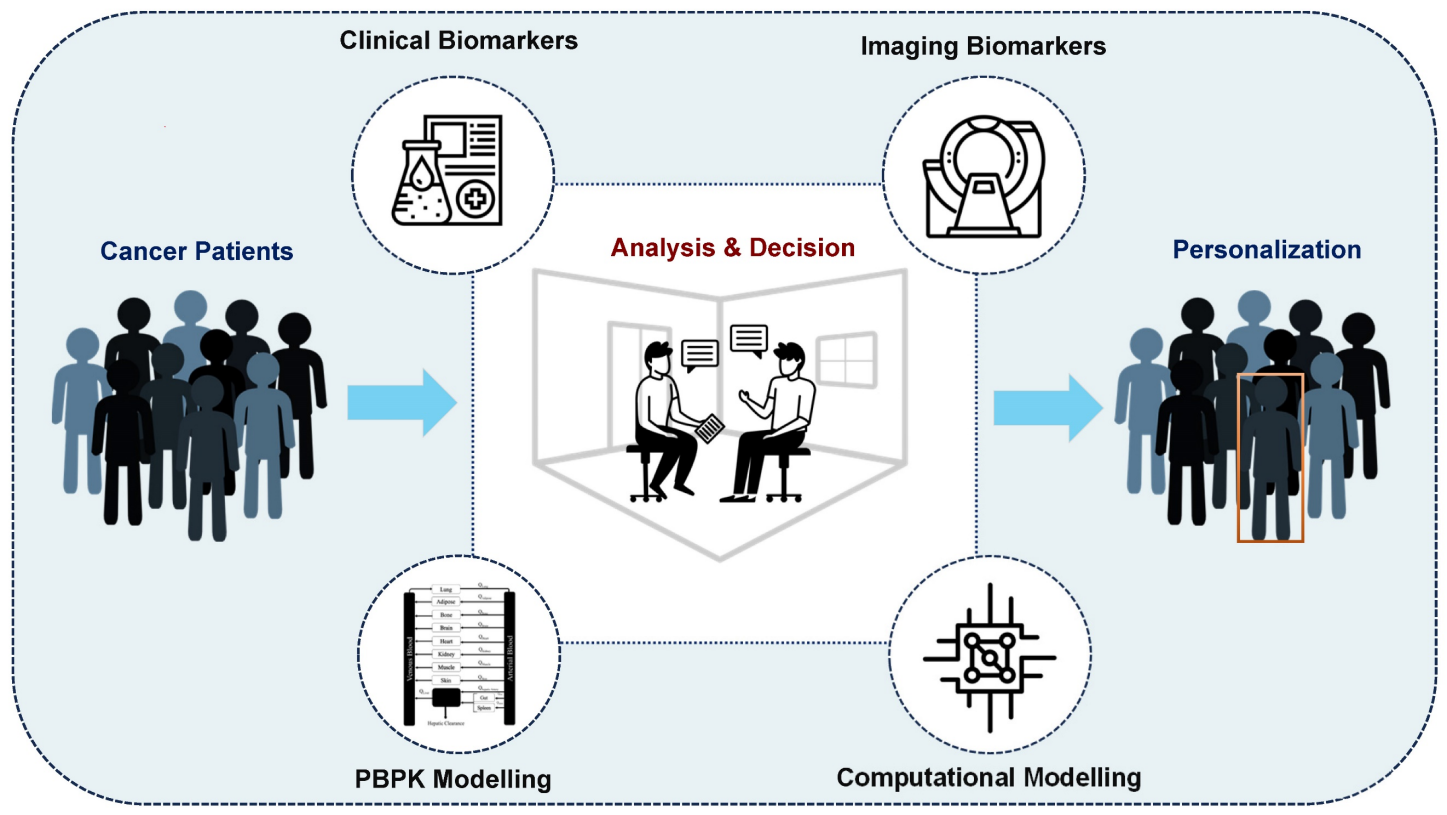
Approaches toward personalized radiopharmaceutical therapies. We divide these approaches into four main categories, including (i) Clinical Biomarker-based, (ii) Imaging Bionarker-based, (iii) Physiologically-based pharmacokinetic (PBPK), and (iv) Computational Oncology-based.

Some initial efforts have already been made toward developing precision RPTs and have yielded promising results. We have categorized and summarized these approaches in **Figure 1**, dividing them into four main categories, including: (i) clinical biomarkers, (ii) imaging biomarkers, (iii) physiologically-based pharmacokinetic (PBPK), and (iv) computational oncology-based methods. However, the potential for personalization of RPTs through these approaches has not yet been fully investigated. Furthermore, many of these methods are population-based and therefore lack personalization 25, while also failing to account for the multi-scale modeling required in RPTs.

Moreover, computational and imaging technology has undergone very rapid and recent change. Substantial improvements in generative AI 26, coupled with improvements in computational technology, can now afford improved simulation and big data analytics to improve the field of mathematical oncology 27. Generative AI provides the capability to generate new or improved images; as an example, there is significant potential to map shorter-scan or lower-resolution images (e.g. SPECT) to higher-resolution (PET) images for improved assessments. Mathematical or computational oncology involves the application of mathematical models and computational approaches to understand and predict the dynamics of cancer growth, progression, and response to treatment. In nuclear medicine, these techniques can be utilized to optimize imaging and therapeutic strategies, enhancing the precision and effectiveness of cancer diagnosis and treatment. Significant advances in scanner technology have resulted in substantial improvements in scanner sensitivity 28,29 and the ability to observe organ-organ interactions in real-time through extended field-of-view systems 30. These advances have also led to the development of novel tools and methods for cancer diagnosis, prognosis, and therapy, including predicting patient outcomes and optimizing treatment protocols 31,32. The development of cancer digital twins (DTs) is a significant example of computational and mathematical oncology 33-35. DTs can apply mathematical models to create virtual replicas of tumors and their microenvironments, which can be used to simulate and predict the behavior of tumors in response to different treatments 36. This allows for more precise and personalized treatment planning and optimization, potentially leading to better outcomes for cancer patients.

In the realm of RPTs, we recently proposed theranostic DTs (TDTs). We predict that TDTs will revolutionize the design and optimization of RPT by, for the first time, enabling realistic simulations of their distribution and effect in a virtual environment 37-39. TDTs could be used to predict the distribution of radiopharmaceuticals in tissues and organs, as well as their radiation dose to both the target and healthy tissues, which can inform the design of optimal radiation dose regimens for RPTs. In addition, TDTs can also be used to personalize treatment planning by simulating the response of patient-specific tumors and tissues to various radiation dose regimens, which can help tailor the treatment to the individual patient. Overall, the use of DTs in RPTs has the potential to improve treatment efficacy and minimize radiation toxicity, ultimately leading to better patient outcomes.

The objective of the present work is to propose a comprehensive roadmap for the development, enhancement, and adoption of TDTs as a promising future strategy for improving RPTs. We first briefly discuss the concept of DTs, TDTs and then present a framework and roadmap for the development and implementation of TDTs. Finally, we discuss our proposed strategy and outline future directions, followed by concluding remarks.

## 2. Digital Twins and Theranostic Digital Twins

Recently, DTs have gained significant attention and adoption due to their potential applications in many areas of research and development, including industries and healthcare 40. DTs are virtual replicas of physical objects, systems, or processes that are created using data from different sources 41. They are designed to simulate the behavior and performance of a real system, allowing for better analysis, optimization, and decision-making 42. In healthcare, DTs have been directed toward developing feasible and more accurate models for personalized diagnosis or therapy 43. Furthermore, a range of DTs are designed or suggested in different scales from subcellular to whole organ levels, and specific or general disorders have been investigated 44. The idea of developing and using DTs to understand tumor dynamics and personalized management of cancer patients has grown in popularity due to improvements in experimental approaches to quantitatively characterize cancer and improvements in the mathematical and computational sciences and modeling strategies 45.

DTs have found various applications in healthcare, including biomanufacturing, viral infections, orthopedic surgery, cardiology, nutrition, drug discovery, neurology, and oncology. DTs have received attention in drug discovery, with the concept of Drug Development Digital Twins (DDDT) proposed for drug research and development 46. As another example, they have also been developed for neurology to improve diagnosis, treatment, and management strategies for multiple sclerosis patients 47. As such, they have been proposed for precision cancer care and have been used to predict the progression of prostate cancer 48 and neurological complications in pediatric cancers 49.

**Figure 2:**
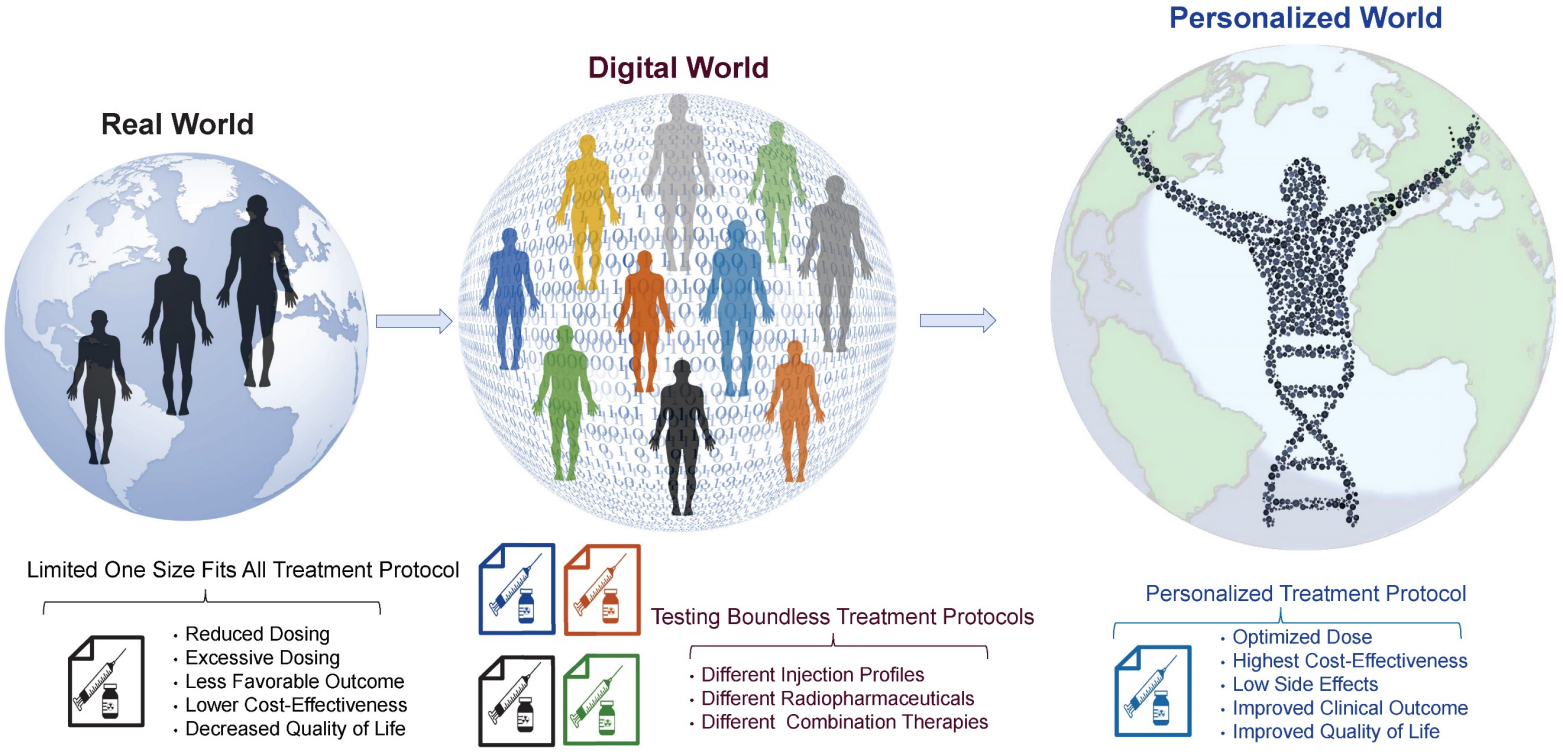
From the real world toward the personalized world through the digital world. In the current real world, patients are treated using “one size fits all” approaches resulting in non-optimal clinical outcomes. Using the digital world, referred to as “theranostic digital twins” (TDTs), different treatment protocols can be developed and tested in a digital environment. The optimal personalized protocol can be utilized for each patient in the “personalized world” which we envisage will result in improved clinical outcomes and quality of life.

Current RPT protocols often overlook several important factors that may influence treatment planning and outcome, including tumor characteristics, such as heterogeneity, tumor microenvironment, spread, size, volume, and location, as well as normal tissue factors like functionality, radiosensitivity, and perfusion 50,51. Moreover, other critical factors, such as immune system status, tumor and normal tissue pharmacokinetics, receptor density/heterogeneity, protein binding, age, sex, socioeconomic status, and combination therapies, are not fully considered in these therapies 52. This limited, impersonalized approach can increase the likelihood of over/underdosing, side effects, and treatment failure. Therefore, we propose a more personalized approach, using theranostic digital twins (TDTs), which leverage patient data and advanced computational modeling to create a virtual treatment world. Here, a range of treatment protocols can be tested and optimized, and personalized protocols can be selected for individual patients. This approach has the potential to improve treatment outcomes and quality of life and reduce patients' costs and economic burden. **Figure 2** provides a visual representation of our proposed approach toward connecting the small physical world to the personalized world using the TDT approach. TDTs are computational avatars for patients based on imaging and clinical data on which virtual treatments can be performed. TDTs will enable optimized and personalized RPTs by simulating injection protocols with acquired data from diagnostic nuclear medicine scans or other imaging modalities.

Our proposed TDT uses available patient-specific information, including imaging and clinical data. The TDT is much more than a specific solution. It is a discovery/solution-providing paradigm providing a tool to personalize RPTs by enabling investigation of a variety of intervention parameters that can be optimized, e.g., optimal injected radioactivities (for a given specific activity), injection sites, injection intervals and profiles, and combination interventions/therapies. TDTs can enable reliable predictive dosimetry, for which we will leverage clinical trial data and prospectively collect additional clinical data to validate and refine our TDTs. We will predict the time activity curves (TACs) of a therapeutic radiopharmaceutical and will perform absorbed dose assessments even before the administration of the first therapy cycle (what we term “predictive dosimetry”, moving beyond our current post-image-acquisition dosimetry).

Our TDT approach is consistent with the important ongoing theme of deploying *in silico*/computational/virtual clinical trials 53,54. This is also reflected in increasing guidance by the United States Food and Drug Administration (FDA) on the use of such models and trials in submissions 55,56, and the European Union REACH (Registration, Evaluation, Authorization, and Restriction of Chemicals) legislation's goal of reducing animal testing by embracing *in silico* methods 57. New legislation for the FDA introduced in 2023 mandates that instead of animal testing, new drugs can now move onto human trials following successful rounds of “non-clinical tests,” which includes “Computer modeling” 58.

## 2. TDTs framework

**Figure 3:**
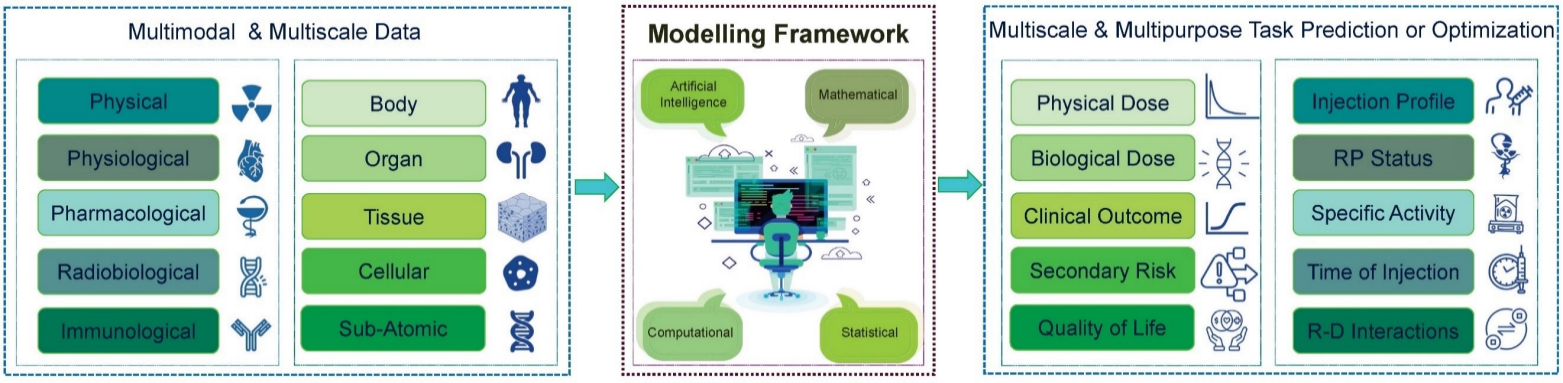
Framework for developing TDTs. In this framework, we need multimodal and multi-scale data, which cover all we need for modeling. These data will be processed and analyzed through advanced mathematical and computational approaches and then will be used to predict and optimize the treatment. The TDTs will be used based on the scale and purpose of the required task. RP: Radiopharmaceuticals; R-D: Radiopharmaceuticals-Drug.

In pursuit of our main objective to develop TDTs for personalized RPTs, we begin by presenting a general framework that outlines how we plan to predict treatment outcomes. This framework provides a comprehensive overview of the necessary steps and components required to predict and optimize treatment plans accurately. We depict our TDT framework in **Figure 3**. As is shown, our TDT models can be developed through advanced computational, mathematical, or artificial intelligence (AI) models using multimodal and multi-scale data. Then, the developed models will be utilized to predict and optimize multi-purpose or multi-scale tasks based on the clinical situation. In the following section, we describe in detail the components of our framework.

### 2.1. Data and data collection strategies

**Figure 4:**
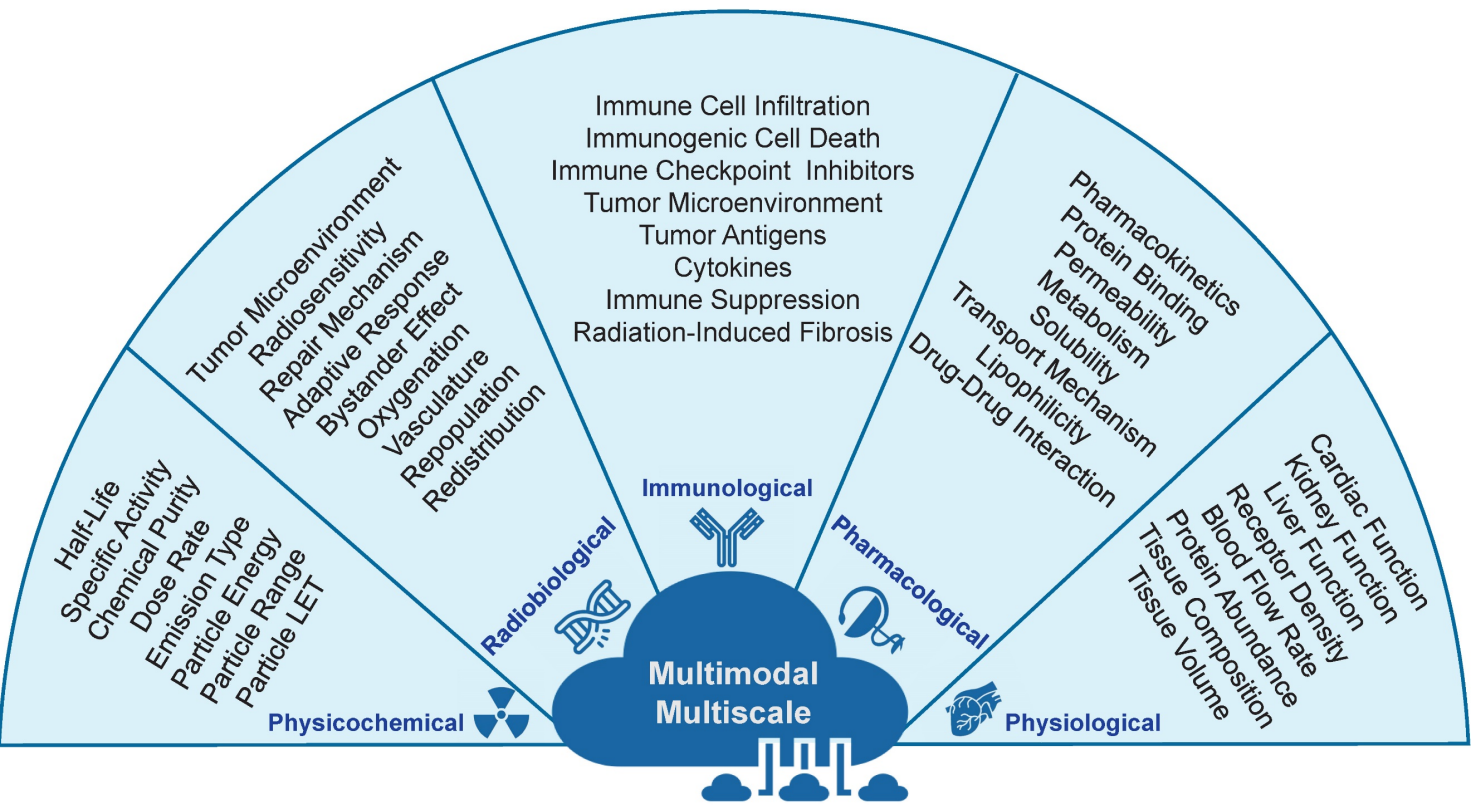
Different types of data are required to develop TDTs. These multi-scale/multimodal data include physical, radiobiological, immunological, pharmacological, and physiological.

RPT is a complex therapeutic approach which operates at the intersection between different branches of sciences, including medicine, physics, mathematics, radiobiology, radiochemistry, physiology, pharmacology, and immunology 59. Incorporating these interdisciplinary approaches will be critical in ensuring that RPTs are effective, safe, and have minimal patient side effects. Furthermore, since the effects of RPTs on the human body can be highly complex and interdependent, the study and optimization of RPTs can be achieved through a multi-scale framework that considers the therapy at different scales. In this case, to develop TDTs, we need multi-scale data from the sub-atomic to whole-body levels. As such, data can be collected and analyzed in a temporal scale from a few seconds to months. From this point of view, it is necessary to gather data that encompasses a range of scales and modes to create versatile TDTs models. The following are examples of the types of data that should be collected. In **Figure 4**, these data are described.

To clarify the data collection process, we employ a comprehensive strategy integrating various diagnostic techniques tailored for this therapeutic context. Essential biochemical markers indicative of the patient's health status are assessed through blood tests, while urine tests provide valuable insights into metabolic processes influenced by the therapy. Imaging modalities, particularly SPECT/CT scans in the case of Lu-177-PSMA, play a pivotal role by offering detailed anatomical and functional information, contributing to a holistic dataset for precise treatment evaluation. Additionally, real-time monitoring is facilitated through the integration of body-attached sensors, capturing dynamic physiological changes over the course of radiopharmaceutical therapy. Given the nuanced nature of this therapeutic approach, the proposed methodology involves a series of targeted experiments and examinations to ensure the comprehensive acquisition of pertinent data. Pharmacokinetics, a critical parameter in RPTs, is meticulously determined through the analysis of both animal and patient data, establishing a robust foundation for our model. This detailed explanation aims to provide a more explicit depiction of the specialized methodologies employed in our data collection process, specifically tailored to the intricacies of RPTs applications like Lu-177-PSMA. It is essential to emphasize that the proposed methodology for data collection endeavors are inherently multiscale and multimodal, spanning across various levels, including population, individual, tissue/organs, and cellular dimensions. Our comprehensive approach involves gathering diverse datasets across these scales. Subsequently, we plan to establish a robust biobank, utilizing the collected data as a foundation. This strategic initiative positions us to develop diverse models and conduct thorough analyses, fostering a deeper understanding of the intricate relationships and patterns inherent in the data collected across different scales.

#### 2.1.1. Physicochemical data

Physicochemical data are integral for developing (TDTs) models, emphasizing radiopharmaceutical physical properties 55. Key considerations include physical half-life, maximum administrable activity, and specific activity, influencing delivery, effectiveness, and toxicity 56,57. Chemical and radiochemical impurities impact in vivo behavior, affecting efficacy and safety 58. Dose rate, emission type, particle energy, range, and linear energy transfer (LET) are crucial factors influencing radiobiological events 59,60. For example, Beta-emitting radioisotopes offer a longer range and greater tissue penetration due to their longer particle pathlength (≤12 mm) and lower linear energy transfer (LET) (;0.2 keV/mm), while alpha-particles have a moderate pathlength (50-100 mm) and high LET (80 keV/mm). These properties shape the interaction between radiopharmaceuticals and cells or tissues, affecting therapeutic efficacy and potential side effects. Distinguishing between beta-emitting radioisotopes and alpha-particles is crucial for understanding radiation dynamics, impacting treatment effectiveness and potential adverse effects.

#### 2.1.2. Pharmacological data

Radiopharmaceutical pharmacokinetics (radiopharmacokinetics) is critical for the success of RPTs and TDTs modeling 61. It involves studying the distribution and elimination of radiopharmaceuticals within the body, determining treatment effectiveness, and assessing adverse side effects, especially in critical organs like the kidneys and bone marrow 62. Important pharmacological factors include protein binding, metabolism, permeability, solubility, transport mechanism, lipophilicity, and drug-drug interactions 63. Blood proteins like albumin significantly impact distribution and elimination 64, while permeability and solubility affect distribution throughout the body. The transport mechanism dictates radiopharmaceutical movement through the body, and lipophilicity impacts distribution 65. Radiopharmaceutical-drug interactions must be considered during TDT development, involving potential interactions with other drugs, influencing treatment effectiveness and safety, especially in combination therapies 21.

#### 2.1.3. Physiological data

Understanding physiological factors influencing radiopharmaceutical pharmacokinetics is crucial for developing and personalizing RPTs. Achieving optimized therapeutic outcomes with minimal side effects requires accurate modeling and personalized dosing, considering various physiological parameters 67-69. Pharmacokinetics encompass absorption, distribution, metabolism, and elimination of radiopharmaceuticals, making it crucial for RPT success. Physiological processes, including organ function, receptor density, blood flow rate, protein abundance, tissue composition, and volume, intricately influence radiopharmaceutical pharmacokinetics. For example, cardiac function is vital for distributing radiopharmaceuticals via blood flow, while kidney function plays a crucial role in elimination through urine. Receptor density dictates radiopharmaceutical binding, and blood flow rate affects distribution to various organs. Protein abundance in the blood can influence metabolism and elimination processes. Advancements in on-chip technology, such as microfluidic chips or organoids, offer promising avenues for enhancing physiological data acquisition 71,72, providing detailed insights into physiological processes and facilitating refinement of RPT modeling and dosing strategies.

#### 2.1.4. Radiobiological data

Developing personalized TDTs for RPTs requires understanding crucial radiobiological phenomena influencing tumor cells and surrounding tissues 73-75. Factors include the tumor microenvironment, radiosensitivity, repair mechanisms, adaptive response, bystander effect, oxygenation, vasculature, repopulation, and redistribution. The tumor microenvironment significantly impacts tumor growth and RPT response, influenced by factors like hypoxia, pH, and nutrients. Radiosensitivity, indicating the intrinsic susceptibility of cells to radiation, must be considered in TDT modeling, emphasizing the need for a deeper integration of radiobiology into nuclear medicine practices 73-77. Key radiobiological factors, such as DNA repair capacity and mechanisms, are pivotal in the modeling process, considering the decreasing dose rate pattern in RPT. This pattern affects biological processes like redistribution, repopulation, and adaptive response, necessitating their incorporation into TDT models 74. Additionally, tumor vasculature and oxygen status are crucial elements for a comprehensive representation of radiobiological complexities in personalized TDTs 75.

#### 2.1.5. Immunological data

Radiopharmaceuticals can activate immunological pathways crucial for RPT success 78. These pathways modulate the immune response in both tumor and normal tissues, affecting clinical outcomes 79. In TDT modeling, considering the immunological status of the tumor microenvironment provides insights into interactions with tumors and normal organs. Critical pathways include the modulation of immune cells, inducing immune cell infiltration, promoting immunogenic cell death, and activating immune checkpoint inhibitors 80,81,82. Considering immune checkpoint inhibitors in TDT modeling helps optimize RPT efficacy. Cytokine expression in the tumor microenvironment modulates the immune response, leading to changes in RPT efficacy. Tumor antigens trigger an immune response, and their consideration in TDT modeling helps optimize RPT efficacy by promoting an immune response against tumor cells. Immune suppression, limiting RPT success, should be considered in the modeling to identify strategies to overcome this barrier.

### 2.2. Modeling

In developing TDTs, advanced mathematical, statistical, computational, and AI approaches are pivotal for creating precise models and optimizing RPTs. Ordinary Differential Equations (ODEs) serve as a mathematical approach to model dynamic biological and physiological systems, including pharmacokinetics and the intricate interactions within cell populations, radiation effects on cell dynamics, and the immune system's response to radiation 60,61. Partial Differential Equations (PDEs) contribute to TDT modeling by capturing the spatiotemporal distribution of radiation in tumors and surrounding tissues, predicting delivered radiation doses, and ensuring treatment effectiveness and safety 62,63. This includes exploring tissue dynamics through Darcy models for fluid flow, comprehensive convection, diffusion, and reaction (CDR) modeling, and histology-driven reaction-diffusion PDE modeling to understand the influence of physiological factors like hypoxia on dose distribution 64-68.

AI approaches, such as machine learning and deep learning, have become instrumental for modeling complex systems by analyzing large datasets, detecting patterns, and revealing intricate relationships that traditional statistical methods might miss 69. Deep learning algorithms, for instance, play a crucial role in analyzing medical images, identifying tumor regions, and optimizing treatment planning and delivery 70. Machine learning extends its application to predict dose distribution based on PET imaging 71,72. Complementary to mathematical and AI-based techniques, computational tools like Monte Carlo simulations simulate radiation behavior at the atomic level, aiding in predicting the biological effects of radiation 73. These collective approaches form a comprehensive strategy for TDT modeling, offering a multifaceted perspective on the spatiotemporal dynamics in biological systems and optimizing RPT outcomes.

### 2.3. Application

Our proposed TDT offers a comprehensive approach to predicting and optimizing the outcomes of RPTs by considering various factors at multiple scales. This model can be used for a range of multi-scale and multi-purpose task predictions or optimization. One of the key predictions our model can make is the physical and biological doses of RPTs. By considering the properties of the radiopharmaceutical, and human body factors, our model could predict the delivered dose to both the tumor and normal tissues. These predictions can inform treatment planning and help ensure that the prescribed dose is appropriate and safe. Another prediction our model can make is the clinical outcome in terms of tumor response, disease progression, and survival. This information can be used to inform treatment decisions and optimize treatment regimens. Secondary risk is another important consideration in RPTs. Our model could predict the likelihood of adverse effects, such as radiation-induced normal organ complications and secondary malignancies. Quality of life is another important consideration in RPTs.

Such a model can predict the potential impact of treatment on the patient's quality of life by considering factors, such as treatment duration, adverse effects, and symptom management. This information can be used to inform treatment decisions and help ensure that the patient's overall well-being is optimized. In addition to predicting outcomes, TDTs can also be used to optimize RPTs. Clinicians and researchers can use our model to optimize injection profiles by determining the optimal amount of injected radioactivity, the number of injections, and the time between injections 74,75. This information can be used to tailor treatment regimens to individual patients and maximize treatment effectiveness. Furthermore, TDTs can be used to optimize specific activity, time of injection, and radiopharmaceutical-drug interactions. By considering the properties of the radiopharmaceutical, the tumor microenvironment, and the patient's medical history and concomitant medications, our model can provide insights into the optimal treatment regimen for each patient.

## 3. TDTs development

A TDT model comprises three main components: a radiopharmacokinetic engine, a radiobiological optimizer, and an immunological modulator. In the following, we describe this model in detail.

### 3.1. Radiopharmacokinetic engine

The main component of our model is a Physiologically Based RadioPharmacoKinetic (PBRPK) model, which can be constructed using mathematical, computational or AI-based approaches. This engine can be developed using physical, pharmacological, and physiological factors through patient data, including imaging, lab tests, and other clinical measurements. The PBRPK has multiple compartments, which are based on the total physiology of the human body. The PBRPK would predict the physical dose and secondary risk and also control the pharmacokinetic and injection profile.

### 3.2. Radiobiological optimizer

A radiobiological optimizer will be added to the model to personalize and optimize our TDT model based on the radiobiological properties. This module captures radiobiological data, such as radiosensitivity, repair, and proliferation capacities using lab tests or other measurements, and RPT can be personalized using this module. Adding this optimizer will improve models to predict biological dose and clinical outcomes.

A range of radiobiological effects, such as cell death, cell repair, cell cycle effect, signal transduction, gene expression, mutagenesis, and genomic instability should be considered. While there has been a multitude of radiobiological studies conducted in the realm of RPTs, a significant amount of information remains elusive. For example, radiogenomics is a rapidly evolving field that aims to identify genetic markers associated with radiosensitivity and response to radiation therapy. By analyzing an individual's genetic profile, radiogenomics can provide valuable information about the likelihood of developing side effects or treatment response to radiation therapy. Radiogenomics holds promise for enhancing RPTs by tailoring treatment plans to individuals' genetic profiles. Understanding how genetic factors impact radiation response can optimize efficacy, reduce side effects, and enhance patient outcomes. However, this field is evolving, necessitating further research to unravel the intricate interplay between genetics and radiation therapy. Continued investigation is essential to integrate radiogenomics data effectively into clinical practice and decision-making.

### 3.3. Immunological modulator

Since the immune system plays a critical role in RPT, our TDT models can be improved using an immunological modulator. This module will add immunological factors, such as tumor microenvironment, immunological cell death, antigen, and cytokine expression on the TDT models to improve and personalize the model. These immune factors can be obtained using lab tests or other measurements***.***

The tumor microenvironment is a complex system that involves a variety of immune cells, growth factors, and cytokines. Understanding the specific characteristics of a patient's tumor microenvironment can help predict their response to different therapies. Similarly, radiation associated immunological cell death; such as apoptosis and necrosis, can also be important predictors of response to therapy. Incorporating antigens into TDT models is also important. Antigens are molecules that are recognized by the immune system and can trigger an immune response. Identifying the specific antigens that are present in a patient's tumor can help predict their response to immunotherapy. Cytokine expression is another important factor that can be incorporated into TDT models. Cytokines are proteins that are produced by immune cells and play a critical role in immune responses and understanding the specific cytokine expression patterns in a patient's immune system can help predict their response to different therapies. Overall, incorporating an immunological modulator into RPT models can provide a more personalized and accurate prediction of a patient's response to therapy. These immune factors can be obtained through lab tests or other measurements, providing valuable information that can be used to optimize patient care.

## 4. TDT roadmap

**Figure 5:**
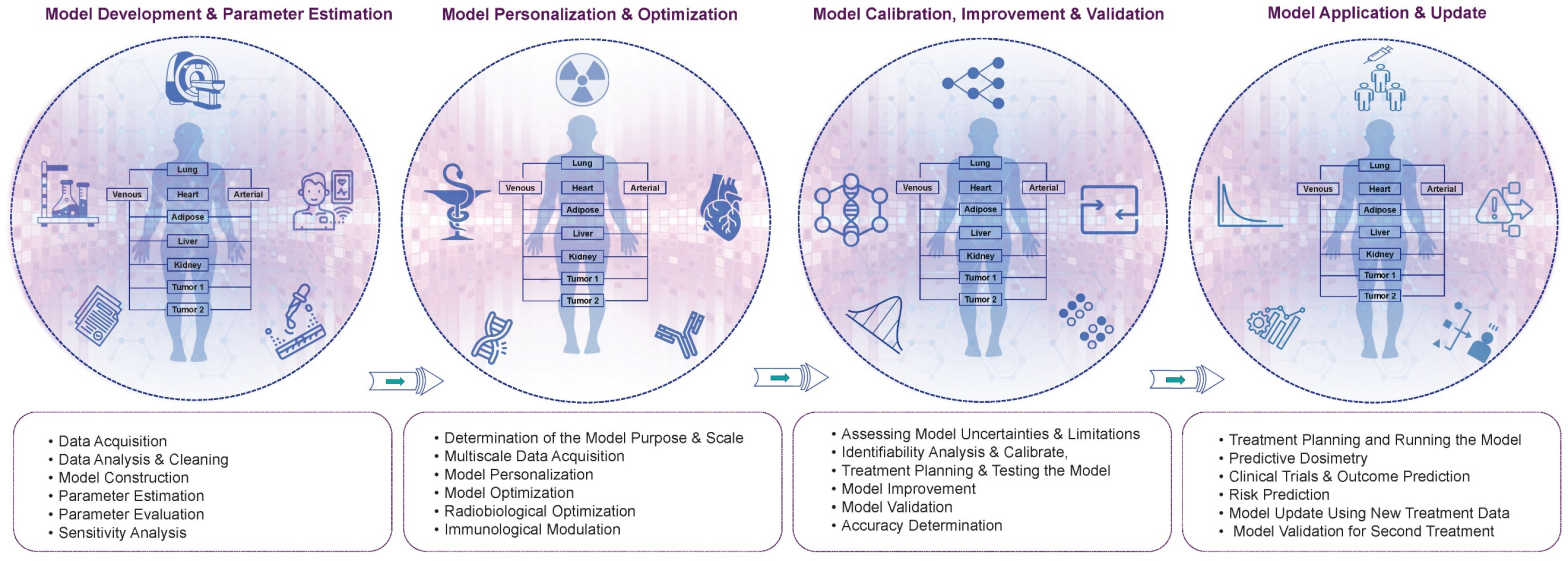
The detailed roadmap for the development of TDTs. We propose four main step processes, including model development, personalization, validation, and application. The specifics of each step are elaborated.

We propose a multi-step approach for the development of TDT, which can be divided into four main steps: (i) model development and parameter estimation, (ii) model personalization and optimization, (iii) model calibration, improvement, and validation, and (iv) model application and update. These steps are outlined in more detail below and in **Figure 5**.

### 4.1. Model development and parameter estimation

The first step in TDT model development is patient data acquisition. As mentioned in the above section, a range of multimodal and multi-scale data is needed to generate the first models. Since our TDT engine is a PBPRK model, the main data are those which impact radiopharmacokinetic. These comprehensive datasets encompass a variety of physical, physiological, and pharmacological information, obtained through precise methods such as blood tests for specific biomarkers, urine tests targeting metabolic indicators, and sophisticated imaging modalities like PET and CT scans to capture detailed anatomical and functional insights. Additionally, wearable body-attached sensors monitor vital signs, providing continuous data on aspects such as heart rate and activity levels. The collection of these data involves a strategic and patient-specific approach. For instance, blood tests may focus on tumor markers, urine analyses may assess metabolic profiles, and imaging modalities may include various scans tailored to individual requirements. Wearable sensors, on the other hand, facilitate continuous monitoring without extensive intrusion, ensuring a holistic representation of the patient's health status.

**Figure 6:**
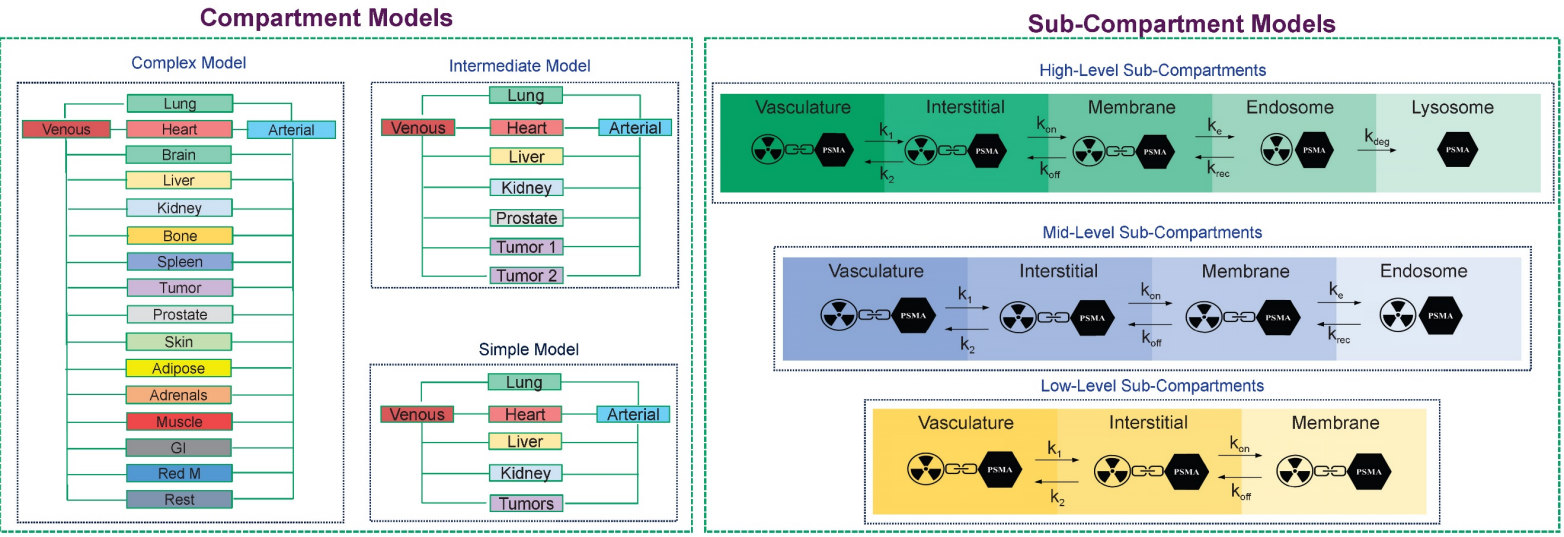
The main core of our TDT model in terms of PBRPK compartment and sub-compartment models. In our TDT modeling approach, based on the patient biology/physiology, radiopharmaceutical type, and clinical needs, different models, from simple to complex PBRPK models, can be developed. At the sub-compartment level, for example, for Lu-177- PSMA therapies, we can have different numbers of sub-compartments which are connected through microparameters, including k_1_, k_2_, k_on_, k_off_, k_e_, k_rec,_ and k_deg,_ which show internalization process of this kind of radiopharmaceutical into a specific tissue including a tumor.

The PBRPK engine, based on the level of personalization/optimization, physiological, and biological characteristics of the patients, as well as the type of radiopharmaceutical, can be made in different modes. For example, it can be made as a complex whole-body model with a multiple organs, compartments, and sub-compartments, or it might be a simple model made of some few compartments. For example, in **Figure 6**, we depict different PBRPK models for Lu-177-PSMA therapies. In this case, the pharmacokinetics of the radiopharmaceutical can be modeled by means of compartment and sub-compartment models of varying complexity 76.

Parameter estimation of the developed PBPRK models is a key step in TDT modeling; it is an inverse problem that can be solved using conventional mathematical methods as well as new AI approaches. For example, conventional methods, such as linear and non-linear least-square fitting 77, genetic algorithms 78, simulated annealing 79, Bayesian 80, and Cluster Gauss-Newton (CGN) methods 81 can be employed. Furthermore, some AI approaches, such as novel biology-informed neural networks (BINNs), can be used to estimate the parameters 82 BINNs have the potential for complete interpretability and the incorporation of explainability algorithms 82. Recent studies have demonstrated the potential of BINNs for stratifying prostate cancer patients according to 83 their treatment-resistance states and assessing the molecular causes of treatment resistance for therapeutic targeting. The goal of BINNs, based on physiologically informed neural networks (PINNs), is to develop a trustworthy and reliable algorithm for parameter inference and prediction of hidden dynamics, a basic topic in systems biology. Inspired by systems biology and incorporating ordinary differential equations into the neural networks, system biology-informed neural networks (SBINNs) may also be deployed. Daneker et al. 84 peer-reviewed reference neural networks informed by systems biology for parameter estimation, checking the possibility of locally identifying parameters and ultimately using SBINNs for parameter identification. While models, such as logistic regression have excellent interpretability, they may have lower predictive performance compared to deep learning models. Additionally, if the network is not properly regularized (as in BINNs and SBINNs), data may be overfitted, and much larger datasets may be needed for generalizable deep learning.

To ensure that the developed models based on their estimated parameters have the highest accuracy and reliability, parameter evaluation and sensitivity analysis should be applied. In this process, the effectiveness of the parameters should be examined based on the relationship between the model's input and output values. Sensitivity analysis is a crucial step to examine how changes in input parameters affect the output of the model 85. This helps to determine which parameters have the most significant impact on the model's output and which are less important. Performing a sensitivity analysis can assist in identifying the parameters with the greatest impact on the model and refining it for improved accuracy and predictive power. Novel methods, such as the use of Fisher-Information-Matrix 86 and Bootstrapping frameworks 87, can be used as sensitivity analysis approaches.

### 4.2. Model personalization and optimization

Improving personalization and optimization is a crucial and significant stage in the development of TDT models. During these stages, the accuracy and effectiveness of the models will be significantly improved. In order to implement these approaches, models need to be enhanced using multi-scale data that include both radiobiological and immunological data. These data types are essential when the goal is to deliver high doses of radiation to tumor tissue while minimizing exposure to surrounding healthy tissues.

Radiobiological data can be incorporated into RPT planning by considering factors, such as repair, repopulation, bystander effect, adaptive response, and radiosensitivity. By incorporating radiobiological data into models, we can develop more accurate and personalized treatment plans that are tailored to the individual needs of each patient. Similarly, immunological data can provide important information about how the immune system responds to cancer cells and to RPTs. By analyzing immune system activity, researchers can identify patients who are likely to respond well to RPTs, as well as those who may require additional treatments, such as immunotherapy.

### 4.3. Model calibration, improvement, and validation

Calibration, improvement, and validation are all important steps in developing and evaluating predictive models. When the first TDT models using PBRPK, optimizer, and modulator are developed, they should be calibrated in terms of adjusting the model's parameters to optimize its performance on a specific treatment planning and scale. Calibration is important because it ensures that the model is tuned to the specific problem at hand and that its predictions are as accurate as possible. The model improvement involves making changes to the model in order to improve its performance or address any limitations or weaknesses. This can involve adding new features or variables, changing the model's structure or architecture, or incorporating new data or knowledge. Model improvement is an ongoing process, as researchers are constantly developing new techniques and approaches to improve the performance of predictive models. Model validation involves evaluating the model's performance on new data or in a real-world setting. The goal of validation is to ensure that the model is accurate, reliable, and effective and that it can be used to make predictions with confidence. TDT models undergo evaluation through various pre-treatment planning tests to identify uncertainties and limitations in these processes. Determining uncertainties and limitations is crucial in refining the model. Improvement opportunities arise at this stage, where incorporating new data and conducting analytical tests can enhance the model's performance.

### 4.4. Model Application and Update

When the modeling process is completed, it can be used for various clinical applications, including treatment planning, Predictive dosimetry, outcome prediction, risk prediction, and running clinical trials. It should be noted that, as the RPTs are delivered through different cycles, the developed TDT models can be applied for the first cycles of therapy, and then they can be updated using these data and improved for the other cycles of therapy, and this process can be repeated. These kinds of dynamic TDTs (DTDTs) will improve RPT personalization and optimization. Models also can be updated using new patient data.

### 4.5. Example of TDTs for personalizing Lu-177-PSMA therapies

**Figure 7:**
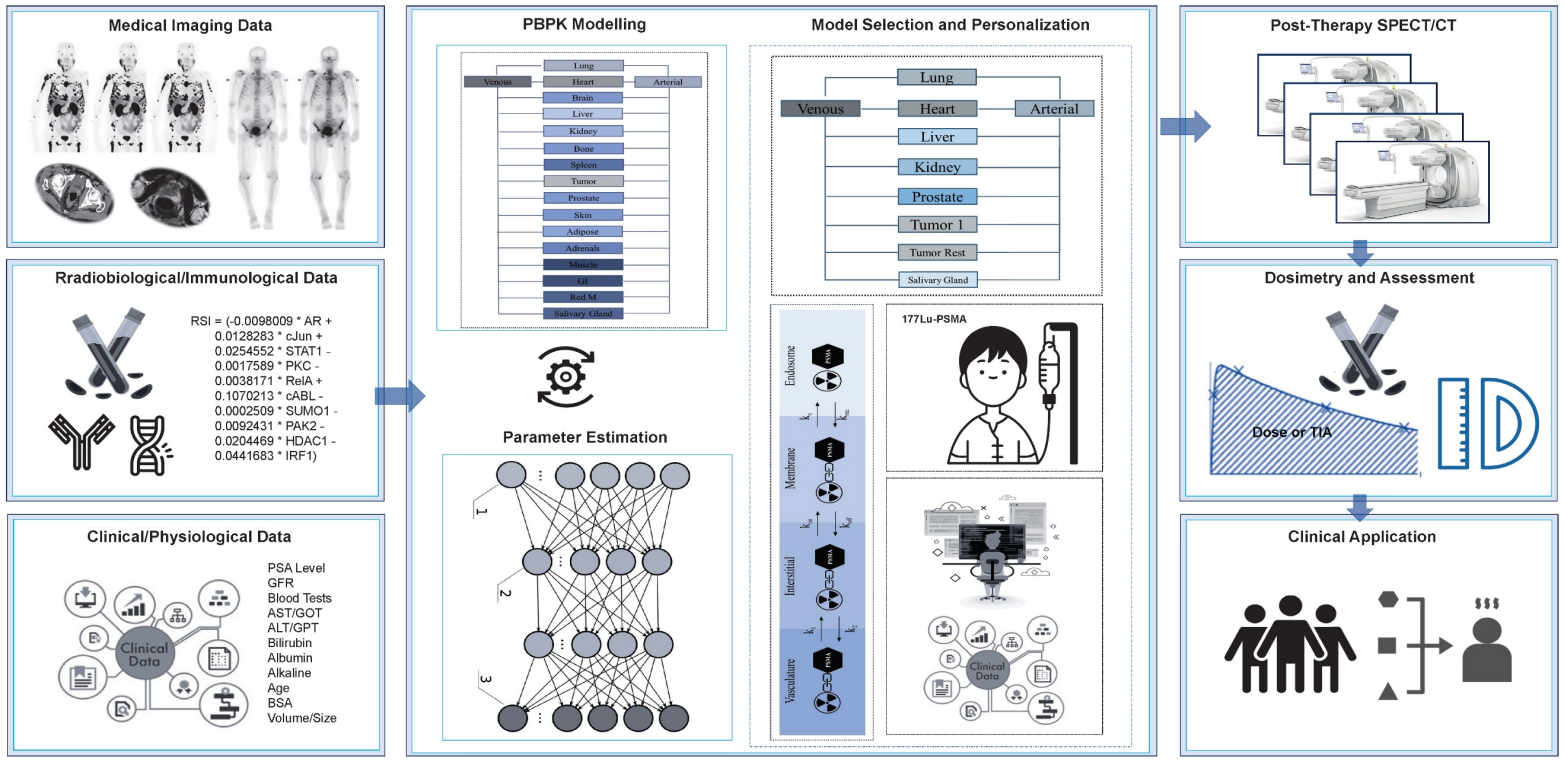
Workflow for designing TDTs for radiopharmaceutical therapies. This case is an example for Lu-177-PSMA therapies.

In the following section, we show how our TDT models could be developed and utilized for patients who receive Lu-177-PSMA as a part of their treatment. We summarize the process in **Figure 7**.

#### Data collection

Consider the scenario where a group of patients has been diagnosed with metastatic castration-resistant prostate cancer (MCRPC), prompting physicians to prescribe Lu-177-PSMA for disease management. As the effectiveness of Lu-177-PSMA hinges on sufficient PSMA receptor expression in lesions, obtaining PET imaging or SPECT data (e.g., Tc-99m-PSMA SPECT/scintigraphy) becomes indispensable for the initial assessment of Lu-177-PSMA treatment planning. In addition to these imaging modalities, other scans like bone scans, CT, and MRI can offer valuable insights into patient characteristics and overall metastatic status, including PSMA-negative lymph nodes and bone metastasis. However, for our model development purposes, a dynamic PSMA-PET/CT scan stands out as it can provide an extensive array of data, encompassing crucial pharmacokinetic parameters. Moreover, considering the availability of PET radiotracers, physicians may choose to order Cu-64-PSMA for PET imaging. This particular radiopharmaceutical, with its extended half-life, has the potential to furnish more comprehensive information regarding the pharmacokinetics of PSMA, contributing to a more detailed understanding of the treatment dynamics. Furthermore, extended fields of view PET scanners, including total body scanners, hold particular significance for dynamic imaging applications.

Using the above-mentioned multimodality images, one will be able to gather data that could be used as preliminary model's inputs. Examples include pharmacokinetic parameters, pattern of time-activity curves (TACs), biodistribution patterns, standardized uptake values (SUVs), anatomical localization, 3D localization, quantification of uptake, anatomical information, tumor size and density, perfusion and diffusion metrics and bone metastasis detection. In addition to the diagnostic images, a wealth of valuable data can be derived from patient documents and a diverse array of examination modalities. Critical information essential for constructing a thorough patient profile includes assessing prostate-specific antigen (PSA) levels and conducting comprehensive kidney and liver function tests. These tests cover parameters such as creatinine, estimated glomerular filtration rate (eGFR), complete blood cell count, as well as liver-specific markers including aspartate aminotransferase (AST/GOT), alanine aminotransferase (ALT/GPT), total bilirubin, albumin, alkaline phosphatase (AP/ALP), lactate dehydrogenase (LD/LDH), and C-reactive protein (CRP). Furthermore, insights into the condition of vasculatures play a pivotal role in this holistic evaluation. This extensive dataset ensures more profound understanding of the patient's physiological profile, fostering a more nuanced and personalized approach in the development of therapeutic strategies.

Additionally, capturing radiobiological and immunological data is essential to grasp the primary characteristics of patients. This information aims to enhance the modulation and optimization of our models. For radiobiological optimization, we can extract blood samples from patients and assess radiosensitivity through genomic analyses such as single nucleotide variations (SNV), copy number variations (CNV), and other pertinent genes in the Radiosensitivity Index (RSI). This index, previously described in literature and is derived from 10 genes (AR, c-JUN [JUN], STAT1, PKC [PRKCB], Rel A [RELA], cABL [ABL1], SUMO1, CDK1, HDAC1, and IRF1). We can then modify our injection prescription based on these findings. On the other hand, given that Radiotherapy-Induced Immune System alterations (RPT) are anticipated, and considering our intention to optimize and personalize the injection dose, it is imperative to gather valuable information on the immune system. This information includes blood count levels, genetic polymorphisms, and the levels of crucial interleukins (e.g., IL-10 and IL-6), NKG2D, KIRs, MHC class I, PD-1, NKG2A, killer immunoglobulin-like receptors (KIRs), as well as chemokine (C-C Motif) ligand 2 (CCL2) and colony-stimulating factor 1 (CSF1).

#### Model development and parameter estimation

In the context of model development and parameter estimation, collected data can be leveraged to construct patient-specific PBPK models. These models encapsulate the unique PSMA pharmacokinetics and the functional attributes of a patient's body, derived from dynamic PSMA PET/CT, complemented by other imaging modalities such as contrast-enhanced CT/MRI and relevant lab tests. PBPK models already integrate, in a knowledge-driven manner 88, the body weight, height, hematocrit level, and tubular extraction rate (TER; used to calculate GFR) influencing compartmental volumes of distribution, blood flow and kidney filtration rate. One may also input tumor and OAR volumes into the models as derived from patient images using an automated segmentation tools 89. The model parameters can then be personalized for individual patients. The process of parameter estimation can be executed through both conventional and AI methodologies, utilizing techniques like least-square fitting and genetic algorithms. Furthermore, other conventional methods like CGN estimation which is a computationally efficient and robust approach can be applied 81. New methods like NNs, neural ODEs and PINNs can more effective and accurate for parameter estimation. For example, Neural ODEs, are useful for dynamic data and can enhance the accuracy and efficiency of pharmacokinetics parameter estimation 90. PINNs also which represent another AI paradigm effective for ill-posed and inverse problems, showing superior capabilities in approximating and generalizing solutions for high-dimensional partial differential equations 69.

#### Model selection, personalization, and application

The approach involves constructing a variety of PBPK models featuring different compartments and sub-compartments. The selection of the most personalized model is based on the individual patient's data, parameters, and fitting results. For instance, model refinement may include simplifying structures to encompass only PSMA-positive organs, grouping all PSMA-negative organs into a single entity with a reduced set of parameters. Conversely, adjustments to the models may be necessary when dealing with large tumors, addressing the potential tumor sink effect, a phenomenon well-documented in clinical observations and supported by modeling simulations.

## 5. Discussion

In recent years, cancer research has shifted towards studying and treating cancer as a systemic disease, which requires multi-scale modeling of cancer diagnosis and therapy 91. Hitherto, research efforts have mainly been focused on the genetic and molecular characteristics of cancer; instead, tumors are complex structures that depend on dynamic interactions between cells and their microenvironments. Cancer systems biology aims to understand the emergent behavior of the malignant system as a whole rather than focusing on individual parts. To analyze and integrate large amounts of data, mathematical and computational modeling are used in addition to standard biological and medical research 92.

In this work, a roadmap is proposed to develop theranostic digital twins aiming at personalizing radiopharmaceutical therapies. Our goal is to enhance patient outcomes. Using the theranostics principle, we can first identify tumor cells expressing specific proteins via diagnostic agents (radiolabeled molecules) and then target those cells using similar molecules labeled with therapeutic radionuclides. As currently implemented, RPTs largely rely on a standardized approach that does not account for inter-patient variations in pharmacokinetics. Our vision for TDTs is to personalize RPT prescriptions in order to maximize radiation doses to tumors while minimizing toxicity to organs at risk (OARs).

TDTs will allow us to personalize dose assessments and predict absorbed doses before patients receive their therapy cycles. These digital twins will incorporate multiple sources of patient data, including clinical history, imaging, and clinical data, to develop predictive models. TDTs will model both pharmacokinetics (how the body processes the drug) and pharmacodynamics (how the drug affects the body). In RPTs, pharmacodynamics involves radiobiology and immunology, making it distinct from chemotherapy, while the unique pharmacokinetics of RPTs differentiates it from external beam radiotherapy (EBRT), presenting unique challenges that TDTs can help address.

In the realm of developing and refining TDTs, the use of statistical models allows for the assessment of relations of interest using data from many patients. The initial step involves harnessing comprehensive population data, which encompasses a broad spectrum of information concerning anatomical structures, physiological responses, immunological, pharmacological, biochemical, and genetic diversity among individuals. This data serves as the cornerstone for constructing a baseline digital representation using mathematical models, which capture distribution and trends within the population. Subsequently, the process transitions into personalization, wherein individual-specific data, including lab tests, medical history, genetics, and previous diagnostic/therapeutics data, is integrated. In this personalized phase, statistical models are applied to analyze and fine-tune the TDT, ensuring a more precise alignment with the unique attributes of each individual. Additionally, thorough validation and calibration of predictions made using data from patient cohorts are emphasized to ensure the reliability and accuracy of the TDT's predictions in real-world clinical settings. By embracing this integrated approach, TDTs are developed to not only reflect the characteristics of the general population but also cater to the personalized intricacies of individual human bodies. Developing highly accurate pharmacokinetic models, specifically PBRPK models, is essential in the process of TDT modeling. Recently, PBRPK models have found much interest in RPTs, and have been used in predictive dosimetry and treatment planning. However, most of these models have relied on population-based parameters, leading to a lack of personalization. Instead we propose the use of advanced approaches, including AI or other inverse problem solution approaches for parameter estimation and personalization.

We expect that the approaches outlined in this roadmap will help improve and personalize TDT models using radiobiological and immunological modules. The radiobiology of RPT has some additional complexities when compared with EBRT; a range of physical parameters, such as particle range, energy, LET and half-life, determine the fate of radiobiological events. In addition, a major critical factor in RPT is dose rate, which has an exponentially decreasing behavior and significantly impacts radiobiological phenomena such as repair, repopulation, and redistribution. Furthermore, the radiation sensitivity of both normal and tumor tissues is a vital factor that should be considered in the model in terms of radiogenomics. Incorporating immunological factors into RPT planning can further improve the accuracy and effectiveness of treatment, ultimately leading to better outcomes for patients. For example, relevant immunological factors associated with the tumor microenvironment should be included in the TDTs for optimal personalization and optimization. Moreover, given that RPT can induce immune effects, such as changes in the expression and activity of T-cells, cytokines, dendritic cells, innate myeloid cells, natural killer cells, and myeloid-derived suppressor cells, it is essential to consider the role of immunology in the TDT modeling process.

One important task for TDT modeling is RPT induced normal organ complications. The dose-limiting organ varies between therapies and is in itself a complex interplay of various physiological, radiobiological and pharmacodynamic factors. For some therapies, such as PRRT, the kidney is the dose limiting organ, where the renal tubules exhibit high uptake resulting in prolonged exposure to radiation with high dose rates 93. For other therapies, such as radioiodine therapy, bone marrow is the dose-limiting organ and, e.g. in prostate cancer RLT, it is the salivary and lacrimal glands 94. Although the dose limiting organ for radioiodine therapy is the marrow, radiation induced sialadenitis can also occur and can be quality-of-life limiting. Understanding the potential for side effects and limiting doses to these organs will help optimize the treatment and improve patient outcomes.

Parameter estimation is a crucial aspect of mathematical modeling, particularly in systems biology, where models of biological reactions often involve many unknown parameters that must be inferred from a limited number of experimental measurements 95. Conventional approaches like least-square fitting, genetic algorithms, and Bayesian methods can be used, but identifying parameters can be challenging due to insufficient data and non-identifiability issues. Structural identifiability analysis, which focuses on identifying the model's parameters, can help overcome these challenges 96. Additionally, SBINNs can be employed for parameter and hidden dynamics identification by incorporating systems of ODEs into the neural networks 97. SBINNs can add further constraints to the optimization of techniques, making it robust to noisy measurements and scattered observations, thus enabling the estimation of unknown parameters based on minimal data. The training process of SBINN should be performed using a loss function that consists of two supervised losses: (i) the discrepancy between the network and measurements and (ii) unsupervised loss (based on ODEs). After parameter estimation by SBINNs, we will consider identifiability (structural and practical) steps to ensure the reliability of the results.

One of the main benefits of TDT is developing a comprehensive scoring system which takes into account multiple factors, including treatment outcomes, normal tissue side effects, quality of life, and cost-effectiveness. This TDT scoring system will enable us to identify the most optimal therapy for each patient and to personalize their treatment plan accordingly. By utilizing this scoring system, we can minimize the risk of side effects on normal organs and maximize the potential benefits of RPT for each patient. Ultimately, this approach can lead to improved treatment outcomes and quality of life for patients undergoing RPT. The use of our TDT models and this scoring system might enable us to perform virtual clinical trials in order to test and understand RPTs better.

Generative AI models hold promise in generating data for both population and individual-based TDTs. For population models, they can simulate various human body scenarios, both normal and abnormal, providing a comprehensive understanding of human physiology. At the individual level, they can generate new data that individual patients may not possess, such as creating pathology maps or simulating pharmacokinetic data from simple anatomical information. Generative AI models can significantly aid in the development of TDTs, particularly in compensating for data scarcity and enabling the simulation of various human conditions, both normal and pathological 98-100. These models excel in creating, manipulating, and diversifying data, thereby enhancing data acquisition, communication, management, digital modeling, and analysis. By synthesizing these methodologies, the development of TDTs for 177Lu-PSMA therapy has the potential to revolutionize personalized cancer treatment strategies, advancing both efficacy and patient outcomes. Drawing inspiration from prior work such as scGen and PathologyGANs 100, novel approaches can be devised to tailor Generative AI techniques for TDTs in the context of treatments like Lu-177-PSMA therapy. For instance, generative models such as Variational Autoencoder (VAE) or Generative Adversarial Network (GAN) models present promising avenues for simulating essential data in TDT modeling for Lu-177-PSMA therapy. As a prospective example, these advanced techniques can accurately emulate crucial factors like cellular uptake and receptor densities, utilizing simulated PET scans and histological images derived from simple anatomical inputs. This not only streamlines treatment planning but also enables precise customization for individual patients, ultimately maximizing the effectiveness and personalization of the therapy.

One important aspect of precision RPTs that should also be considered in our modeling is microdosimetry. Microdosimetry plays a crucial role in evaluating the impact of radiopharmaceuticals, especially with alpha emitters, such as Actinium 225, on the subcellular and DNA levels 101. The decay scheme of these alpha emitters is instrumental in performing microdosimetry, allowing for the estimation of damage produced, such as the calculation of double breaks in DNA 102. Additionally, in microdosimetry, it is vital to account for the position of the radioactive source (radiopharmaceutical) within different parts of the cell, including the cell membrane, cytoplasm, and nucleus, as well as considering the position of the target. Furthermore, parameters like the percentage of cellular uptake of the radiopharmaceutical are integral to microdosimetry and warrant inclusion in our modeling efforts. Therefore, a comprehensive consideration of these factors enhances the precision and personalization of dose prescriptions in our RPT plans.

Longitudinal data, acquired both before and during RPTs, will play a pivotal role in the continuous improvement and refinement of TDTs. The advantages of longitudinal data will lie in its ability to capture changes over time, offering insights into the dynamic nature of a patient's condition and treatment response. This information is essential for updating TDTs to enhance their predictive accuracy and effectiveness. Longitudinal data can be obtained through various means, including biosensors, wearable devices, such as detectors, imaging techniques, and other innovative technologies. While these data sources provide valuable information, their collection and analysis present challenges. The process is often costly, time-consuming, and may induce patient discomfort due to the need for regular monitoring. Striking a balance between obtaining comprehensive longitudinal data and minimizing patient burden remains a critical challenge. As technology advances and methodologies evolve, optimizing data collection methods and addressing associated limitations will be instrumental in leveraging the full potential of longitudinal data for refining TDTs and improving patient outcomes in the realm of RPTs.

However, we should emphasize that developing TDTs might have challenging issues in terms of acquiring patient data, developing models, estimating parameters, and addressing ethical considerations. Since cancer is a complex disease and also RPT mechanisms are not fully investigated, the interaction of radiopharmaceuticals with this system, their pharmacokinetic and pharmacodynamic, should be studied and analyzed through advanced modalities. Despite the challenges, TDTs offer a promising research framework for studying RPTs in an *in-silico* ecosystem. To effectively integrate TDTs into clinical practice, a thorough and collaborative approach involving researchers, medical physicists, technologists, and clinicians is essential. This approach must adapt to the inherent complexity of various clinical settings, patient conditions, and the required level of optimization and personalization.

The process begins with comprehensive data collection, drawn from diverse sources such as clinical trials, lab-based biomarker analyses, and generative AI approaches. These data are then carefully organized into TDT banks within specific timelines, facilitating comprehensive model development. Modelers utilize this data to establish initial models, which are further refined through iterative personalization using patient-specific information. However, the degree of personalization may vary based on patient circumstances; for example, in cases of complex conditions, the focus may shift from personalization to addressing immediate treatment needs. Additionally, while prioritizing user-friendly software for smooth integration into clinical workflows is crucial, the final decision-making process ultimately lies with clinicians, physicists, and patients. This underscores the importance of collaborative decision-making tailored to individual patient needs and clinical requirements, ensuring the effectiveness and appropriateness of treatment strategies guided by TDTs across diverse clinical settings.

Capturing the complexity of the human body necessitates the use of various data types, often multi-dimensional in nature. These data types include clinical data, patient-derived information, biometrics, medical history, multi-omic data, and medical images. The foundational aspect of constructing digital twins lies in the collection of high-volume, high-quality data. However, the process of gathering and storing this information can be intricate and challenging. Challenges further arise due to differences in data type, veracity, volume, and availability, all of which should be carefully considered when building digital twins with multi-dimensional datasets. To ensure accuracy and consistency, standardizing data collection protocols is critical. Digital twins, in particular, require continuous, real-time, and ongoing multi-dimensional dynamic data collection to accurately reflect the current state of patients and adapt to changes over time.

For efficient analysis and modeling, establishing databases to store and organize this vast array of data, along with robust connection systems between patients and digital twins, is imperative. Data diversity is essential for creating generalizable and fair models during development. Furthermore, patient privacy and security are paramount considerations in the realm of digital twins, demanding safeguards against unauthorized access and breaches. Additionally, the dynamic nature of patient movement during treatment introduces a challenge in data acquisition, necessitating a harmonization of data sources to maintain the integrity of information. Addressing these intricacies will be crucial steps to advance the field of digital twins in healthcare.

We also should mention that the determination of an appropriate sample size for the development of TDTs holds paramount importance and is intrinsically linked to the intricate nature of our modeling approach. The scale of our model development, influenced by factors such as the incorporation of diverse imaging and testing modalities, the degree of personalization and optimization sought, and the specific algorithms or models employed for parameter estimation, necessitates a nuanced discussion on sample size considerations. In an ideal scenario, where comprehensive multi-center, multi-modality, and multiscale data are readily available, a larger sample size could significantly enhance the robustness and generalizability of our TDTs. However, acknowledging the real-world constraints and variability, we aim to convey that, in certain instances, meaningful TDTs can still be constructed even with a more limited number of patients, emphasizing the adaptability and potential of our approach across varying data availabilities and model complexities

## 6. Conclusion

Present-day RPTs are generally performed using a “one size fits all” approach. The emerging framework and technology of digital twins, as we elaborate in this work, presents a new framework for the personalization and optimization of RPTs. Our proposed roadmap for developing TDTs is an achievable strategy that can be used to improve the delivery of RPTs. TDTs can be developed using patient data and can be personalized through accurate parameter estimation and model validation. By developing personalized TDTs, we can ensure that each patient receives optimal treatment that is tailored to their individual needs. This approach has the potential to improve treatment outcomes, reduce side effects, and enhance the quality of life for patients undergoing RPTs. Overall, the integration of TDTs offers potential for advancements in certain aspects of the field of RPTs and may enhance patient care. Meanwhile, there is a need to carefully assess and validate reliability of predictive results. It is also crucial to assess the feasibility of implementing and integrating TDTs in clinical practice, considering the need to maintain efficient and streamlined protocols within hospital settings.

## Abbreviations

AI: Artificial Intelligence
BINN: Biology Informed Neural Network
CD: Cluster of Differentiation
DDDT: Drug Development Digital Twins
DT: Digital Twins
DTDT: Dynamic Theranostic Digital Twins
eGFR: Estimated Glumoral Filteration Rate
FDA: Food and Drug Administration
GBq: Giga Becquerel
HCC: Hepatocellular Carcinoma
LET: Linear Energy Transfer
MIBG: Metaiodobenzylguanidine
mABs: Monoclonal Antibodies
NET: Neuroendocrine Tumor
ODE: Ordinary Differential Equation
PBPK: Physiologically Based Pharmacokinetic Modeling
PBRPK: Physiologically Based Radiopharmacokinetic
PCa: Prostate Cancer
PINN: Physics Informed Neural Network
PSMA: Prostate-Specific Membrane Antigen
PVNS: Pigmented Villonodular Synovitis
PSA: Prostate Specific Antigen
REACH: Registration, Evaluation, Authorization, and Restriction of Chemicals
RPT: Radiopharmaceutical Therapy
SBINN: System Biology Informed Neural Network
SIRT: Selective Internal Radiation Therapy
TAC: Time Activity Curve
TDT: Theranostic Digital Twins
